# Rho-dependent termination and RNase E-mediated cleavage: dual pathways for RNA 3′ end processing in polycistronic mRNA

**DOI:** 10.1128/jb.00437-24

**Published:** 2025-02-27

**Authors:** Heung Jin Jeon, Monford Paul Abishek N, Xun Wang, Heon M. Lim

**Affiliations:** 1 Cancer Research Institute, Chungnam National University 26715 , Daejeon, Republic of Korea; 2 Department of Biological Sciences, College of Biological Sciences and Biotechnology, Chungnam National University 26715 , Daejeon, Republic of Korea; 3 National Key Laboratory of Agricultural Microbiology, College of Life Science and Technology, Huazhong Agricultural University 47895 , Wuhan, People's Republic of China; The Ohio State University, Columbus, Ohio, USA

**Keywords:** mRNA 3′ end, exoribonuclease digestion, RNase E-mediated cleavage, Rho-dependent transcription termination, polarity

## Abstract

“Pre-full-length” transcripts are produced at the end of the polycistronic galactose (*gal*) operon, 5′ *galE-galT-galK-galM 3′*, via Rho-dependent transcription termination (RDT) and -independent transcription termination. The 3′ end of the full-length *galETKM* mRNA is acquired by exonucleolytic processing of the 3′-OH ends of the pre-full-length transcripts. However, the *gal* operon produces an mRNA termed *galE* whose 3′ end forms approximately 120 nucleotides downstream of the *galE* stop codon, within the subsequent gene, *galT*, thereby establishing polarity in gene expression. In this study, we investigated the molecular processes that generate the 3′ end of *galE* mRNA. We discovered that the 3′ ends of pre-galE mRNA are produced in the middle of *galT* as a result of the combination of two separate molecular processes—one previously reported as RDT and the other as unreported RNase E-mediated transcript cleavage. The 3′ ends of pre-*galE* mRNA undergo exonucleolytic processing to the 3′ end of *galE* mRNA observed *in vivo*. A hairpin structure containing an 8 bp stem and a 4-nucleotide loop, located 5–10 nucleotides upstream of the 3′ ends of *galE* mRNA, blocks exoribonuclease digestion and renders transcript stability. These findings demonstrate that RNase E—contrary to its general role in mRNA degradation—produces RNA 3′ ends that regulate polarity in gene expression.

**IMPORTANCE:**

This study reports the findings of two molecular mechanisms that generate the 3′ ends of pre-*galE* mRNA in the *gal* operon, viz., Rho-dependent transcription termination and RNase E-mediated cleavage. These 3′ ends are subsequently processed to produce stable *galE* mRNA with a hairpin structure that prevents exoribonuclease degradation. This mechanism establishes gene expression polarity by generating the 3′ end of *galE* mRNA within *galT* in contrast to the usual mRNA degradation role of RNase E. The study reveals a unique role of RNase E in mRNA processing and stability.

## INTRODUCTION

In *Escherichia coli*, transcription initiated from the promoter is terminated by two transcription termination mechanisms, Rho-dependent transcription termination (RDT) and -independent transcription termination (RIT) (1). In principle, the termination process generates the 3′ end of the transcript, the final nucleotide containing a free 3′OH group (1). However, this nascent 3′ end is not necessarily the mature RNA’s final form (1). Instead, the 3′ end undergoes immediate exonucleolytic digestion (3′ to 5′), which is blocked by a nearby hairpin structure that confers stability to the mature mRNA (2, 3).

**Fig 1:**
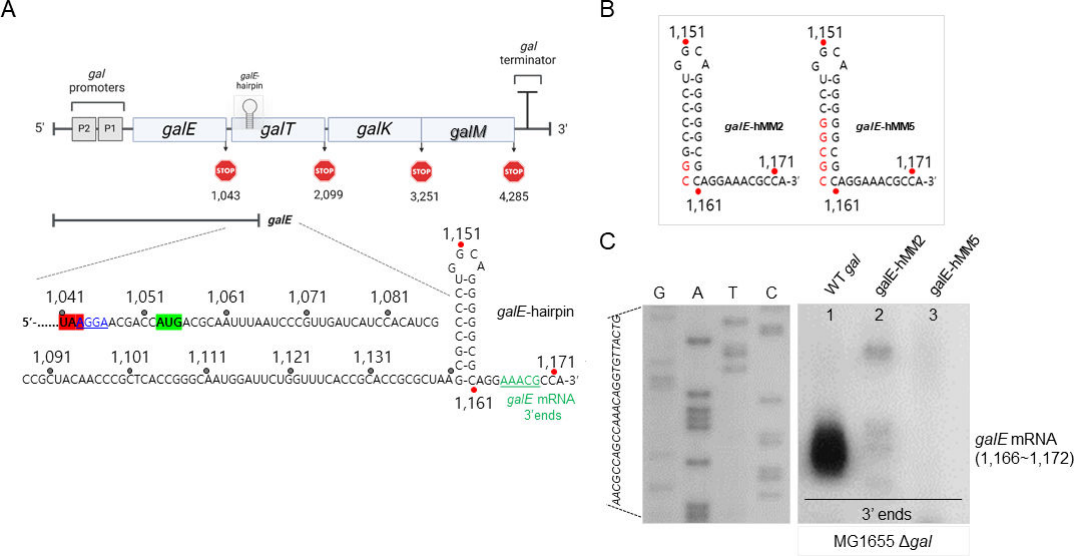
A hairpin structure, *galE* hairpin, is responsible for the functioning of the 3′ end of *galE* mRNA as an exo-block. (**A**) Galactose operon (top) and nucleotide sequence (bottom) of the 3′ end of *galE* mRNA (green) in the WT *gal* operon. The *galE* stop codon is highlighted in red, and the *galT* initiator codon and SD sequence are highlighted in green and blue (underlined), respectively. The *galE* hairpin structure is depicted based on base complementarity between positions 1142 and 1161. Numbers indicate the nucleotide residue coordinate of the *gal* operon, which starts from the transcription initiation site of the *galP1* promoter. (**B**) Schematics of *galE*-hMM2 and *galE*-hMM5 mutants. The base changes are in red. The *gal* mutants were generated in the single-copy plasmid, pGal, where the entire *gal* operon is cloned and assayed in MG1655 cells from where the entire *gal* has been removed, MG1655Δ*gal*. (**C**) 3′ RACE assay of *galE* mRNA 3′ ends from MG155 cells harboring the plasmid-borne *galE*-hMM2 and *galE*-hMM5 mutant operons. The *galE* mRNA 3′ ends (1166–1172) are missing in lanes 2 and 3. DNA sequencing ladders that serve as length markers are in lanes marked G, A, T, and C.

Transcription initiated from the *P1* and *P2* promoters of the *gal* operon terminates at the end of the operon, generating the full-length mRNA referred to as *galETKM*, which harbors the open reading frames of the following four structural genes of the operon: *galE*, *galT*, *galK*, and *galM* (4, 5). The terminator hairpin serves as the Rho-independent transcription terminator, doubling as a structural barrier to 3′-to-5′ exoribonuclease digestion, thereby stabilizing the 3′ end of *galETKM* mRNA (2, 6). Transcriptions that go through the terminator hairpin are terminated at the Rho-dependent terminator; Rho-terminated transcripts are rapidly processed by exonuclease digestion (2, 7). Thus, transcription terminates at RIT or RDT in the *gal* operon (2, 6, 8). The *gal* operon produces not only the full-length mRNA but also three additional mRNA species, *galETK*, *galET*, and *galE*, possessing 3′ ends at the end of each gene, *galK*, *galT*, and *galE*, respectively (Fig. 1A) (5, 8–10). The sRNA, Spot 42, binding at the intercistronic sequence of *galT-galK* of mRNA causes RDT and RNase E-mediated transcript cleavage (11–13).

The production of these *gal* mRNA species, *per se*, establishes polarity in gene expression, causing higher expression of promoter-proximal genes than distal genes (5, 14, 15). This polarity is rooted in the molecular processes generating the 3′ ends of these mRNA species (5, 7, 13). In this study, we demonstrate that, at the 3′ end of the smallest mRNA species from the *gal* operon, the *galE* mRNA is generated by two different mechanisms, viz., RDT (previously reported) (7) and an unreported RNase E-mediated transcript cleavage. We discovered that RNase E-mediated transcript RNA cleavage also generates the 3′ end of *galE* mRNA. These findings indicated that, in contrast to the typical role of RNase E in mRNA degradation, RNase E-mediated transcript cleavage might result in the formation of the 3′ end of mRNA (11). This challenges the conventional view that transcription termination is the sole method for forming mRNA 3′ ends and suggests that RNase E-mediated cleavage may control polarity in the *gal* operon.

## RESULTS

### The 3′ end of *galE* mRNA is processed from those of two “pre-*galE*” mRNA, which is blocked by a hairpin structure

The *galE* mRNA appears to be approximately 1.2 kb in a northern blot when probed with the E-probe that hybridizes to the first half of *galE* (5). The 3′ end of *galE* mRNA is generated at 1166–1171 (the *gal* coordinate starts from the first nucleotide of the *P1* transcript) approximately 130 nucleotides downstream from the stop codon of *galE* and approximately 120 nucleotides downstream from the initiator codon of *galT* (Fig. 1A) (7). Analysis of the RNA secondary structure suggested that at the 3′ ends of *galE* mRNA, a stem with six consecutive G:C and 1 U:G base-pairings and a loop with four nucleotides could be formed at 5–10 nucleotides upstream from the major 3′ ends of *galE* mRNA (1166–1172) (Fig. 1A) (7).

Nucleotide changes in the DNA of the *gal* operon were achieved in a single-copy plasmid, pGal, where the entire *gal* operon is cloned (see Materials and Methods). We analyzed the resulting *gal* mutants in MG1655 cells from where the entire *gal* has been removed, MG1655Δ*gal* (9). We removed two base pairs from the bottom of the stem of the *galE* hairpin by changing C1161 and G1160 to their complementary nucleotides, i.e., guanine and cytidine, respectively (Fig. 1B). The resulting *gal* mutant, gal-*EHMM2*, failed to produce 3′ ends at 1166–1172 (lane 2 in Fig. 1C). We further removed five base pairs from the stem by changing the nucleotides from 1156 to 1161 to their complementary nucleotides (Fig. 1B). The resulting mutant, gal-*EHMM5*, also failed to produce the 3′ end at 1166–1172 (lane 3 in Fig. 1C). These findings demonstrated that the *galE* hairpin is a critical factor for generating the 3′ end of *galE* mRNA at 1166–1172. The role of the *galE* hairpin probably is to block exoribonuclease digestion that is initiated somewhere downstream and render stability to the mature mRNA (7).

To locate the genetic loci where the exoribonuclease digestion initiates molecular processes that generate the 3′ ends of *galE* mRNA, we inserted an additional *galE* hairpin at 1,200 and generated a *gal* mutant, double hairpin at 1,200 (*DH1200*), where the second hairpin starts at 1,200 and ends at 1,219 (7). The results of the 3′ Rapid Amplification of cDNA Ends (RACE) assay of the *DH1200* mutant demonstrated that the 3′ ends of *galE* mRNA decreased by ~50% ± 2% of that of WT (Fig. S1) (7). Interestingly, we observed that this decrease in the 3′ ends of *galE* mRNA was because the 3′ ends at 1169–1172 (upper 3′ ends) are not produced (Fig. S1). However, in the *DH1200* mutant, we did observe a new cluster of 3′ ends at 1219–1223, 5–11 nucleotides downstream of the foot of the stem of the inserted *galE* hairpin (Fig. S1) (7). These findings suggest that the second hairpin reduced the 3′ ends of *galE* mRNA by half the 3′ ends of *galE* mRNA, possibly by blocking the 3′ to 5′ exonuclease digestion downstream of position 1200 where the hairpin was inserted. In the *DH1200* mutant, the production of the upper 3′ ends at position 1169–1172 was absent, whereas a new production of the 3′ ends at position 1219–1223 was observed. This finding suggests that the 3′ ends of *galE* mRNA are generated through RNA processing from two separate pre-*galE* mRNAs, each having 3′ ends located at different positions, viz., (i) pre-*galE1* having 3′ ends between 1171 and 1200 and (ii) pre-*galE2* having 3′ ends downstream of 1200.

**Fig 2:**
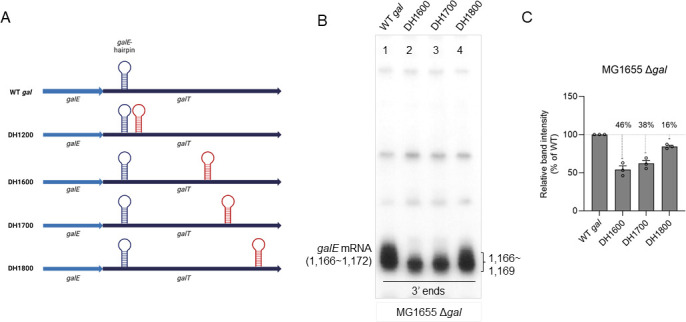
Exoribonuclease digestion initiates molecular processes that generate the 3′ ends of *galE* mRNA. (**A**) Schematics of double hairpin (DH) mutants: *DH1200*, *DH1600*, *DH1700*, and *DH1800*. The inserted hairpins are in red. (**B**) 3′ RACE assay of *galE* mRNA 3′ ends from MG155 cells harboring the plasmid-borne *gal DH1600*, *DH1700*, and *DH1800* mutant operons. The presence of residual RNA secondary structures that cause slower migration may be the cause of larger bands above the *galE* mRNA 3′ ends. (**C**) Relative band intensity of the *galE* mRNA 3′ ends for the 3′ RACE assay from (**B**). Error bars represent the mean fold-change ± standard deviation from three independent experiments.

To locate the 3′ end of pre-*galE2*, we inserted the *galE* hairpin at 1600, 1700, and 1800 positions, generating *DH1600*, *DH1700*, and *DH1800*, respectively (Fig. 2A). We analyzed the 3′ end of *galE* mRNA at 1166–1172 in these mutants. When the second *galE* hairpin was located 400 nucleotides downstream of 1200 (*DH1600*), the 3′ end of *galE* mRNA decreased by 46% ± 8% with an absence of production of the upper 3′ ends at position 1169–1172 (lane 2 in Fig. 2B). When the second *galE* hairpin was located 500 and 600 nucleotides downstream of 1200 (*DH1700* and *DH1800*), the 3′ end of *galE* mRNA decreased by 38% ± 7% and 16% ± 3%, but there was a gradual increase in the 3′ end of *galE* mRNA at 1166–1172 (Fig. 2B and C). Its formation reached ~54% ± 8% of WT in *DH1600* and ~62% ± 7% of WT in *DH1700*. It increased to ~84% ± 3%, nearly that of WT in *DH1800* (lanes 3 and 4 in Fig. 2B and C). The 3′ RACE assay did not detect these 3′ ends because they are probably subjected to 3′ to 5′ exonucleolytic digestion, which is blocked by the *galE* hairpin to generate the 3′ end of *galE* mRNA.

### RNase E-mediated transcript cleavage and RNase II processing of terminated RNA generate the 3′ end of pre-*galE2*

**Fig 3:**
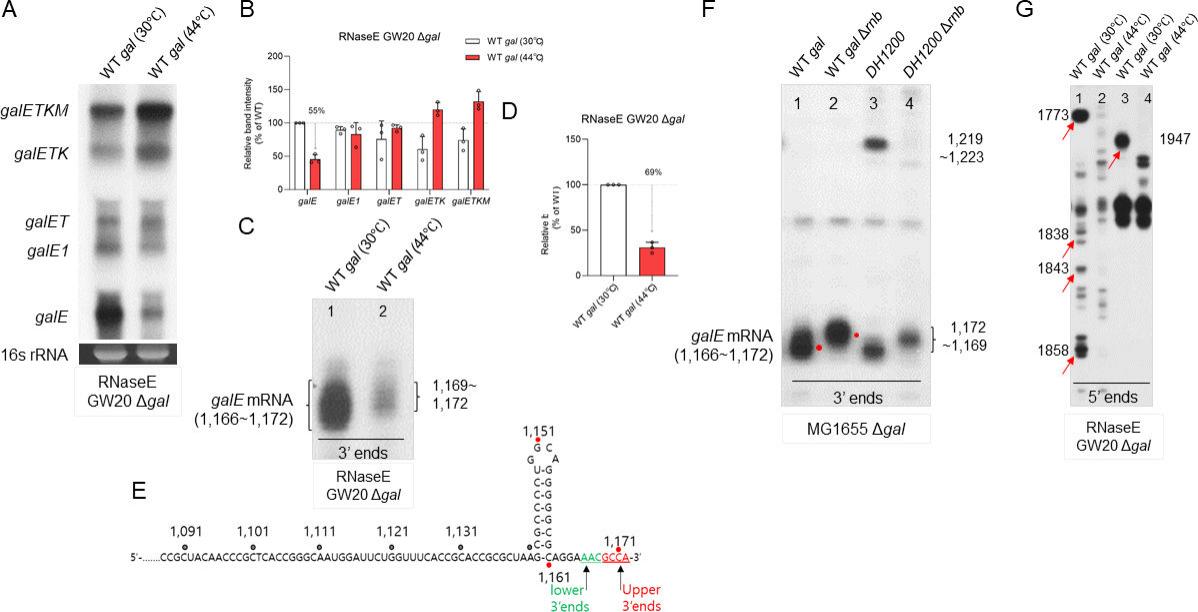
RNase E-mediated endonucleolytic cleavage and RNase II-mediated exonucleolytic processing are the source of the 3′ ends of pre-*galE*. (**A**) Northern blot with the E probe of the *gal* mRNA from GW20Δ*gal* (temperature-sensitive RNase E mutant) cells. Northern blot of E DNA probe (500 bp) was used for the assay that was generated by polymerase chain reaction (PCR) amplification with primers corresponding to the *galE* region (from +27 to +527 in gal coordinates), subsequently radiolabeled with 32P as previously described. Shown at the bottom is 16S rRNA, which was used as a loading control. (**B**) Relative band intensity of *galE* mRNA for the northern blot assay from (**A**). Error bars represent the mean fold-change ± standard deviation from three independent experiments. (**C**) 3′ RACE assay of the 3′ ends of *galE* mRNA from GW20Δ*gal* (temperature-sensitive RNase E mutant) cells. Cells were cultured at both permissive (30°C) and nonpermissive (44°C) temperatures for analysis. (**D**) Relative band intensity of *galE* mRNA 3′ ends for the 3′ RACE assay from (**C**). Error bars represent the mean fold-change ± standard deviation from three independent experiments. (**E**) Nucleotide sequence of the upper (red) and lower (green) 3′ ends of *galE* mRNA based on the 3′ RACE assay from (C). (**F**) 3′ RACE assay of *galE* mRNA 3′ ends from Δ*gal*Δ*rnb* cells*,* where RNaseII is deleted from the chromosome harboring the plasmid-borne *gal DH1200* mutant operon. The red dot shows the shift in the 3′ ends in the absence of RNase II. (**G**) 5′ RACE assay of *gal* mRNA to identify RNase E cleavage from GW20Δ*gal* cells. The lanes show the 5′ ends of RNA at the permissive (30°C, lanes 1 and 3) and the nonpermissive (44°C, lanes 2 and 4) temperatures between 1701 and 1971. The red arrow represents the 5′ ends that are absent at the nonpermissive temperature.

We previously reported that RDT occurs at the end of *galE* in the *gal* operon (7). Experiments conducted using the Rho inhibitor bicyclomycin (BCM) demonstrated that RDT generates the 3′ ends of pre-*galE1* (1,183) (7). We hypothesized that an endoribonucleolytic cleavage on a *gal* transcript could generate the 3′ end of pre-*galE2*. Therefore, we anticipated that the *galE* mRNA would decrease in the precise endoribonuclease mutant cells like RNase E, as it is the major endoribonuclease in *E. coli* (16–18). To determine if this nuclease is responsible for producing the 3′ ends of pre-*galE2*, we investigated its presence in the temperature-sensitive GW20 (*ams1ts*) strain for RNase E activity (17). Our northern blot assay revealed that the amount of *galE* mRNA decreased by ~55%± 7% at the nonpermissive temperature (44°C) compared to that at the permissive temperature (30°C) (Fig. 3A and B). In contrast, there was no significant reduction in the levels of other *gal* mRNAs (Fig. 3A and B), which might be because RNase E cleaves the polycistronic *gal* operon transcript in a manner that destabilizes *galE* mRNA along with stabilizing other components, such as *galETK* and *galETKM*, resulting in increased levels of downstream mRNAs. The 3′ RACE assay revealed that the 3′ ends of *galE* mRNA decreased by ~69% ± 6% that of WT; importantly, only the upper 3′ ends at 1169–1172 were formed (Fig. 3E), suggesting that the 3′ end of pre-*galE2* could be generated by the cleavage of transcript RNA by RNase E (Fig. 3C and D).

RNase II aids in the degradation of RDT-generated mRNA 3′ end in the *trp* operon of *E. coli* and bacteriophage T3 (19). We used the *ΔgalΔrnb* strain (RNase II-deleted) (2) with both the WT and DH1200 variant *gal* plasmids to explore this phenomenon. Interestingly, our 3′ RACE assays of the *gal* transcripts revealed that the 3′ ends at position 1166–1172 shifted to 1169–1172 (upper 3′ ends) in the WT *gal* (Fig. 3F), and the 3′ ends at position 1166–1169 shifted to 1169–1172 in *DH1200* (inserted second hairpin), with the 3′ ends at 1219–1223 disappearing (lanes 3 and 4 in Fig. 3F). Based on these results, we hypothesized that post-RDT events downstream indicate that an unidentified RNase probably processed the Rho-terminated 3′ ends from 1183 to 1169–1172, and RNase II would then have removed the final three nucleotides from the 3′ ends, changing it from 1169–1172 to 1166, thus generating the final stable 3′ ends of *galE* mRNA at 1166–1172 (Fig. 3E). This finding also confirms that in *DH1200* (inserted second hairpin), the 3′ ends at 1219–1223 could be a result of RNase E-mediated transcript cleavage where the second hairpin blocked the 3′ to 5′ exonuclease digestion of the 3′ ends of “pre-*galE2*.”

As RNase E cleavage can cause changes in the abundance and integrity of RNA transcripts, understanding where and how RNase E cleaves the transcripts will help decipher the posttranscriptional control mechanisms. For determining the exact location of the 3′ ends of pre-*galE2*, we investigated the 5′ ends of RNA between positions 1700 and 1900, rather than directly searching for the 3′ ends. We performed a 5′ RACE assay on total RNA extracted from the GW20Δ*gal* strain harboring the pGal plasmid. At the permissive temperature (30°C), several 5′ ends were identified through the RACE assay (lane 1 in Fig. 3G). Remarkably, at the nonpermissive temperature (44°C), these 5′ ends were absent (lane 2 in Fig. 3G). These findings suggest that RNase E cleaves the *gal* transcript in an endonucleolytic manner at these positions *in vivo*. Moderate sequence specificity is associated with RNase E cleavage, which is characterized by a consensus sequence, 5′-R(A/G)NW(A/U)UU-3′ (R = A or G, *N* = any nt, W = A or U) (18, 20), where RNase E cuts between residues N and W, resulting in the W residue at the 5′ end (18). DNA sequence analysis indicated the presence of consensus sequences of RNase E-cleavage sites between positions 1700 and 1900.

The *in vivo* and sequence analyses demonstrated that RNase E cleaves the *gal* transcript residues between positions 1700 and 1900, resulting in 5′ ends at positions (lane 2 in Fig. 3G). These findings not only support the idea that RNase E-mediated cleavage generates the 3′ end of pre-*galE2* but also reveal that aberrant cleavages by RNase E downstream of 1700 reduce the 3′ end of *galE* mRNA at 1166 and 1172. By identifying the cleavage sites, the 5′ RACE assay provides insights into how RNase E affects the stability and maturation of RNA transcripts, such as the *galE* mRNA, which is critical for clarifying the mechanisms of gene regulation at the RNA level. These findings are consistent with those of previous experiments (Fig. 2) and suggest that inserting a hairpin every 100 nucleotides into the system exposed RNase E cleavage sites, which improved RNA stability and accumulation over time by blocking the 3′ to 5′ exonucleolytic digestion blocked by the inserted hairpins, resulting in a gradual increase in *galE* mRNA levels. Therefore, the potential instability from RNase E cleavage is outweighed by the stabilizing effect of inhibiting exonuclease activity.

### Both the RNase E-mediated transcript cleavage and RDT regulate *galE* mRNA 3′ end *in vivo*

**Fig 4:**
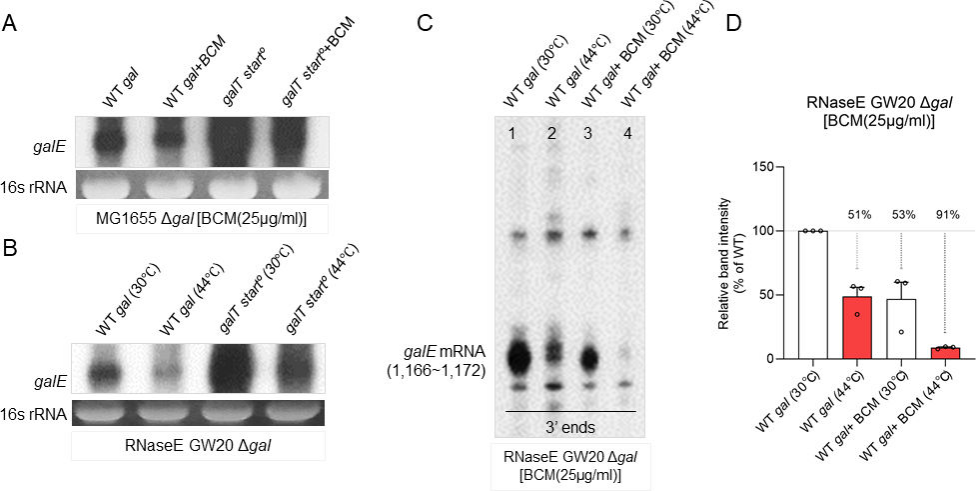
Rho and RNase E affect *galE* mRNA. (**A**) Northern blot with the E probe of *gal* mRNA from MG1655 cells harboring the plasmid-borne *galT* start° mutant operon with or without the Rho inhibitor bicyclomycin (BCM). To determine whether RDT produces *galE* mRNA, we treated MG1655 cells (OD600 of 0.6) in LB medium with 25 µg/mL BCM for 10 min. (**B**) Northern blot with the E probe of *gal* mRNA from GW20Δ*gal* cells harboring the plasmid-borne *galT* start° mutant operon. (**C**) 3′ RACE assay of *galE* mRNA 3′ ends from GW20Δ*gal* cells with or without the Rho inhibitor BCM. Inactivation of RNase E eliminates the “lower bands,” as it processes RNA. Conversely, inhibiting Rho disrupts transcription termination, leading to the accumulation of unprocessed or terminated RNA, which corresponds to the loss of “upper bands.” This distinction provides a compelling basis for differentiating the two 3' ends (see Fig. 3E). (**D**) Relative band intensity of *galE* mRNA 3′ ends for the 3′ RACE assay from (**C**). Error bars represent the mean fold-change ± standard deviation from three independent experiments.

At the *galE–galT* junction, the stop codon of *galE* and the initiator codon of *galT* are separated by nine nucleotides. To investigate the effect of removing *galT* translation initiation on transcription, we changed the initiator codon of *galT* (AUG) to an ordinary codon (AAA), generating a *gal* mutant (*galT* start*°*) for northern blotting. The northern blot of *galE* mRNA in the *galT* start*°* revealed an extremely thick and smeared RNA band starting at 2.0 kb (lane 3 in Fig. 4A) down to the beginning of the operon and a diminished full-length *galETKM* and *galET* (Fig. S2A). These findings show that most of the *gal* transcription in the *galT* start*°* mutant terminated downstream, and a few transcription events reached the end of the operon. Based on these data, we suggest that RDT and possibly the degradation of the 3′ ends of Rho-terminated transcript mRNAs are the cause of the thick and smeared band in the *galT* start*°* mutant. We treated the *galT* start*°* mutant with BCM (Rho inhibitor) for 10 min and found a decrease in the thick and smeared band (lane 4 in Fig. 4A), showing that the thick and smeared band, indeed, was caused by RDT. These data suggest that the absence of *galT* translation initiation caused a break in the transcription–translation coupling, which, in turn, caused RDT.

We next performed a northern blot assay on the GW20 (*ams1ts*) strain to explore the impact of RNase E-mediated cleavage of the *galT* start*°* mutant on *galE* mRNA expression. At the permissive temperature (30°C), a distinct band corresponding to *galE* mRNA was observed (lane 1 in Fig. 4B). In the *galT* start*°* mutant, at 30°C, the *galE* mRNA band was significantly increased, indicating increased expression of *galE* mRNA in the mutant strain under the permissive temperature (lane 3 in Fig. 4B; Fig. S2B). At the non-permissive temperature (44°C), the *galE* mRNA band was significantly diminished (lane 2 in Fig. 4B). In the *galT* start*°* mutant*,* at 44°C, the *galE* mRNA band reduced (lane 4 in Fig. 4B) compared with that at 30°C (lane 3 in Fig. 4B), indicating substantial repression by the RNase E of *galE* mRNA (Fig. S2B). These findings suggest that the absence of *galT* translation initiation could also cause RNase E-mediated transcript cleavage.

However, the source of the break-in the transcription–translation coupling in the *galT* start*°* mutant is probably rooted in the translation termination at the stop codon of *galE* (7, 21). We assumed that the transcription–translation break could be prevented if the translation termination of *galE* were removed from the *galT* start*°* mutant. Continuous translation without interruption by termination at the stop codon of *galT* could eliminate the break in transcription–translation coupling. To test this assumption, we changed the stop codon of *galE* (TAA) to an ordinary codon (AAA) in the *galT* start*°* mutant, generating a double mutant *galET-one-frame* for northern blotting. As anticipated, we found that the thick and smeared band indicative of RDT disappeared, and the *galETKM* band was restored to the level of WT (lane 4 in Fig. S3). These findings demonstrate that continuous translation activity prevented the break in transcription–translation coupling. These data also suggest that if the translation termination of *galE* is removed from WT cells, the amount of the 3′ ends of *galE* mRNA at 1166–1172 should decrease because the continuous translation activity at the *galE–galT* cistron junction would prevent the break-in transcription–translation coupling, thereby preventing the occurrence of RDT.

Data from this study suggest that multiple RNase E cleavages occur between 1700 and 1900, and the 3′ end generated at 1183 by RDT is processed to the 3′ ends of *galE* mRNA at positions 1166–1172. Therefore, if we analyze the 3′ end of *galE* mRNA at 1166–1172 in the GW20 (*ams1ts*) strain in the presence of BCM (Rho inhibitor), we anticipate that at the non-permissive temperature (inhibiting RNase E), the 3′ ends at 1166–1172 would significantly reduce. Without BCM, GW20 cells generated approximately half the 3′ ends (~51%) at positions 1166–1172 at the nonpermissive temperature (lane 2 in Fig. 4C). This finding supports the previous finding that RNase E is responsible for producing half the 3′ ends at these positions. When BCM was added, GW20 cells produced approximately half the 3′ ends (~53%) at positions 1166–1172 at the permissive temperature (lane 3 in Fig. 4C), reinforcing the previous result that Rho contributes to the generation of the other half of these 3′ ends. However, in the presence of 40 µg/mL BCM, the 3′ RACE assay of the 3′ ends at 1166–1172 was seldom detected at the non-permissive temperature (lane 4 in Fig. 4C) with a significant decrease of 91% ± 1% (lane 4 in Fig. 4C). When we analyzed the 3′ end of *galE* mRNA at 1166–1172 in the GW20 (*ams1ts*) strain in the presence of *galE* stop° (Rho inhibitor) (7, 21), similar results were observed in the 3′ RACE assay (Fig. S3A and B). This finding supports the assumption that the 3′ ends at 1166–1172 in WT cells originate from two distinct sources, with both RDT and RNase E-mediated cleavage contributing equally to their formation.

## DISCUSSION

### Translation initiation failure and regulation of mRNA 3′ ends

#### Stochastic failure of translation initiation of the leading ribosome evokes RDT

**Fig 5:**
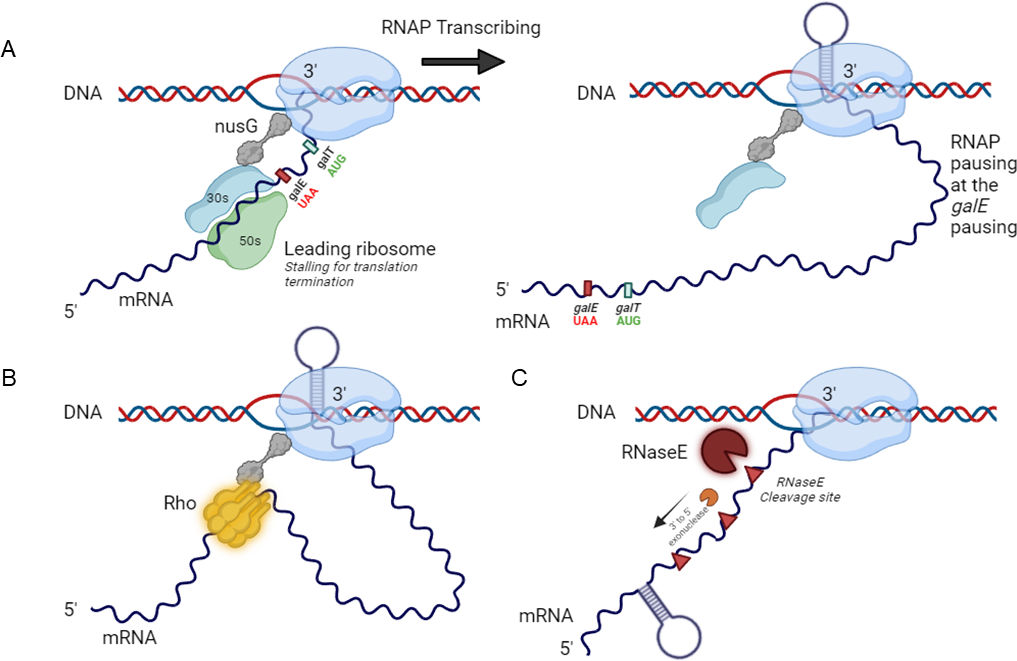
Transcription model. (**A**) Model representing a transcription–translation complex in which the leading ribosome’s 30S remains linked to the paused RNA polymerase prolonged by the *galE* hairpin following the cessation of *galE* translation termination. In a paused state, NusG-S10 might help maintain the position of RNAP on the DNA, ensuring that transcription resumes correctly without disassociating or misprocessing the transcript. (**B**) Model of transcription termination in which the RNA polymerase is stalled downstream of the *galE* stop codon, and Rho is brought to it by the NusG protein. (**C**) RNase E-mediated cleavage model, in which RNase E cleaves the transcript’s RNA free from the ribosome and subjects it to 3′ to 5′ exonucleolytic digestion, which is blocked by the *galE* hairpin to produce the 3′ end of *galE* mRNA.

In *E. coli*, transcription and translation are closely coupled processes (22–28). Ribosomes can bind and initiate translation of the nascent mRNA as soon as the RNA polymerase synthesizes it. When translation begins, the first ribosome on the mRNA is considered the leading ribosome (Fig. 5A). The nascent mRNA will not be adequately shielded by ribosomes if the leading ribosome cannot initiate translation (7, 29). Rho protein binds to the mRNA at specific sites known as Rho utilization (rut) sites (2, 30–34) (Fig. 5B). When ribosomes translate the mRNA, they prevent Rho from reaching these locations by physically obstructing the mRNA (22, 31, 35). In case the process of translation initiation is unsuccessful, the unoccupied mRNA is then available to Rho, which proceeds to unravel the RNA–DNA hybrid in the transcription bubble, ultimately resulting in the termination of transcription (7, 36, 37).

In the *gal* operon, transcription is generally continuous; however, approximately 10% of transcription terminates prematurely at the end of *galE* due to RDT (Fig. 5A and B) (7, 8). This termination is affected by whether the translation of the next gene, *galT*, is successfully initiated. The failure to initiate the translation of *galT* after the completion of *galE* translation results in RDT at the *galE–galT* cistron junction (Fig. 5B) (7). The sequences of nucleotides at the junctions of cistrons might have evolved to enable random failures in the initiation of translation, which could affect the proportion of mRNA generated for various genes in an operon (7, 38).

#### Stochastic failure of translation initiation can also lead to RNase E-mediated transcript cleavage

In the second instance, when translation initiation fails stochastically, in the absence of a bound ribosome, certain regions of mRNA, particularly those near the ribosome-binding site and the start codon, remain unprotected, exposing the RNase E cleavage sites (39). RNase E functions as an endoribonuclease that involves triggering mRNA degradation by cleaving particular sites in the RNA molecule (3, 16, 40). When translation initiation by the ribosome does not occur, these unprotected regions become open to RNase E (39).

In this study, using northern blotting and RACE assays, we demonstrated that RNase E-mediated cleavage contributes to the formation of *galE* mRNA. RNase E cleaves downstream of the *galE* hairpin, facilitating the decay process by allowing 3′→5′ exoribonuclease access for digesting the remaining RNA (Fig. 1, 3D and 5C) (19). This cleavage ensures the complete degradation of mRNA, contributing to efficient RNA processing. We propose that RNase E plays a pivotal role in mRNA synthesis rather than its degradation, as its cleavage activity facilitates the processing steps that enhance the production of *galE* mRNA. Instead of simply breaking down RNA, RNase E-mediated cleavage promotes the generation of mature *galE* mRNA, thereby increasing its abundance and availability for protein synthesis.

For *galETKM* mRNA, its levels increase in RNase E mutant cells when grown at the non-permissive temperature (44°C) (Fig. 3A). This suggests that mRNA stability factors play a role in modulating natural or mutational polarity within the *gal* operon. Considering the role of RNase E, it is plausible that its mutation could cause changes in mRNA stability and, consequently, polarity (5). At the non-permissive temperature (44°C), the altered activity of RNase E potentially affects its impact on polarity, suggesting that the RNase E mutant partially releases natural or mutational polarity in the *gal* operon at this temperature (Fig. S3) (5). This study indicates that in *E. coli*, the gene downstream must start the translation process for transcription to continue beyond intercistronic junctions. If this translation initiation does not occur, it causes the cessation of transcription, which can be facilitated by either Rho or RNase E. These data emphasize the multipart relationship between transcription and translation in the regulation of bacterial genes and provide novel possibilities for understanding operon dynamics and mRNA stability.

### Regulation of mRNA 3′ ends in the polycistronic *gal* operon

The generation of the 3′ end of RNA by RDT and RNase E cleavage is a prevalent mechanism in the formation of *galE* and *galETKM* mRNA within the *gal* operon (Fig. 5). The *gal* operon is essential for the metabolism of galactose in *E. coli* and includes genes that are transcribed from a shared promoter (Fig. 1A). UDP-glucose 4-epimerase is an enzyme involved in the galactose metabolic pathway that is encoded by *galE* in the *gal* operon. By converting UDP-galactose into UDP-glucose, GalE significantly contributes to the utilization of galactose, incorporating it into central metabolism and supporting vital cellular processes (41–43). The production of a stable *galE* mRNA, which encodes the UDP-galactose 4-epimerase, is critical for galactose metabolism (44). For biosynthetic pathways that depend on UDP-glucose, such as glycogen synthesis, stabilizing *galE* mRNA may help sustain a larger and more stable level of UDP-glucose. In the case of *galETKM* mRNA, RDT and RNase E cleavage produce a polycistronic mRNA that includes *galE* along with *galT*, *galK*, and *galM*, encoding enzymes that convert galactose into glucose 1-phosphate. These coordinated mechanisms of 3′ end generations are crucial for the regulation and efficient expression of the *gal* operon, enabling the cell to effectively manage and utilize galactose.

## MATERIALS AND METHODS

### Bacterial strains, growth conditions, and primers

The *E. coli* strains MG1655 Δ*gal*, MG1655Δ*gal*Δ*rnb* (RNase II deletion) (2), and GW20 (*ams1ts*; RNase E temperature-sensitive mutant) (9, 10, 21, 39) were used in this study. Strains of *E. coli* with chromosomal deletions were generated using phage Lambda Red-mediated recombineering MG1655 (45). BCM was a generous gift from Max E. Gottesman (Columbia University, USA). The primers used in this study are listed in Table S1.

### Plasmids

The construction of the pHL1277 plasmid involved the insertion of the galactose operon, spanning from position −75 to +4,333, between the *EcoR*I and *BamH*I sites of the pCC1BAC vector (Epicenter Biotechnologies, USA). The galactose operon was amplified from genomic DNA using the polymerase chain reaction (PCR) primers listed in Table S1. For site-directed mutagenesis, custom synthetic primers containing the specific desired mutations were designed for PCR amplification. After the amplification of DNA fragments containing different mutants of the galactose operon by PCR from the pHL1277 plasmid, these fragments were used as “mega primers” for the next round of PCR. The resulting PCR fragments were then digested with EcoRI and HindIII and subsequently ligated into pHL1277. This process resulted in the generation of derived plasmids, including pHL1751 (EHMM2), pHL1754 (EHMM5), pHL1930 (DH1200), pHL1931 (DH1600), pHL1932 (DH1700), pHL1933 (DH1800), pHL1657 (*galE* stop° mutant), pHL1658 (*galT* start° mutant), and pHL1939 (*galET*-ONE-frame).

### Total RNA extraction and northern blot analysis

Total RNA was extracted from 2 × 10^8^ *E. coli* cells using the Direct-zol RNA MiniPrep kit (Zymo Research) as described previously (9, 39). For the northern blot assay, the RNA samples were resolved by gel electrophoresis and transferred overnight to positively charged nylon membranes (Ambion, USA; TurboBlotter, Whatman, UK). The nylon membranes were hybridized and washed according to the manufacturer’s recommendations (Ambion, United States) (9, 39). The relative intensity of the RNA bands was calculated using the ImageJ software (NIH).

### RACE

The RNA preparation was treated with Turbo DNase I (Thermo Fisher Scientific, USA) to eliminate any DNA contamination. 3′ RACE and 5′ RACE assays were conducted according to previously described methods to amplify either the 3′ or 5′ RNA ends (46–49). The relative intensity of the RNA bands was calculated using the ImageJ software (NIH).

## Data Availability

All data regarding the study is available in the manuscript and its supplementary materials file.

## SUPPLEMENTAL MATERIAL

Supplemental material jb.00437-24-s0001.pdf Figures S1 to S4, Table S1, and Supplemental figure legends.

ASM does not own the copyrights to Supplemental Material that may be linked to, or accessed through, an article. The authors have granted ASM a non-exclusive, world-wide license to publish the Supplemental Material files. Please contact the corresponding author directly for reuse.
