# Supplementary material for: Rho-dependent termination and RNase E-mediated cleavage: dual pathways for RNA 3′ end processing in polycistronic mRNA
Source: J Bacteriol. 2025 Feb 27;207(3):e00437-24. doi: 10.1128/jb.00437-24 (PMC11925234; doi:10.1128/jb.00437-24)
Supplement: Supplemental material — Figures S1 to S4, Table S1, and Supplemental figure legends. [file jb.00437-24-s0001.pdf]

## SUPPLEMENTARY FIGURE AND TABLE LEGENDS

**Figures S1.** 3′ RACE assay of *galE* mRNA 3′ends from MG1655 cells harboring the plasmid-borne *gal DH1200* mutant with an additional E-hairpin at 1200 mutant operons (lane 2). The E-hairpin (42 nucleotides; 1137–1178) at position 1200 in the WT *gal* operon was inserted to generate the double-hairpin *gal* mutant DH1200 (1). Related to Figure 2.

**Figure S2.** Failure of *galT* translation initiation causes RDT. A) Northern blot with the E probe of *gal* mRNA from MG1655 cells harboring the plasmid-borne *galT* start$^{o}$ mutant operon with or without the Rho inhibitor bicyclomycin (BCM). To determine whether RDT produces *galE* mRNA, we treated MG1655 cells (OD600 of 0.6) in LB medium with 25 µg/mL BCM for 10 min. Northern blotting was performed to analyze the total RNA from the cell culture (see *Materials and Methods*). Related to Figure 4A. B) Northern blot with the E probe of *gal* mRNA from GW20Δ*gal* cells harboring the plasmid-borne *galT* start$^{o}$ mutant operon. Cells were cultured at both permissive (30°C) and nonpermissive (44°C) temperatures for analysis. Related to Figure 4B.

**Figure S3.** Northern blot with the E probe of *gal* mRNA from MG1655 cells harboring the plasmid-borne *galE* stop$^{o}$, *galT* start$^{o}$, and *galE-T* ONE frame mutant operons. A slight increase in the other *gal* mRNAs longer than *galE* in the *galE stop$^{o}$* mutant could have resulted from the fact that more transcription might have gone downstream of the 3′ end of *galE*.

**Figure S4.** Continuous translation without interruption by termination at the stop codon of *galT* could eliminate the break in transcription–translation coupling. The 3′ RACE assay compares the WT and *galE* stop$^{o}$ mutant operon from GW20Δ*gal* (temperature-sensitive RNase E mutant) cells, where the entire *gal* operon is deleted from the chromosome. Cells were cultured at both permissive (30°C) and nonpermissive (44°C) temperatures for analysis. B) Relative band intensity for the 3′ RACE assay from (A).

**Table S1**. Primers used in this study and usage.

**Fig. S1**

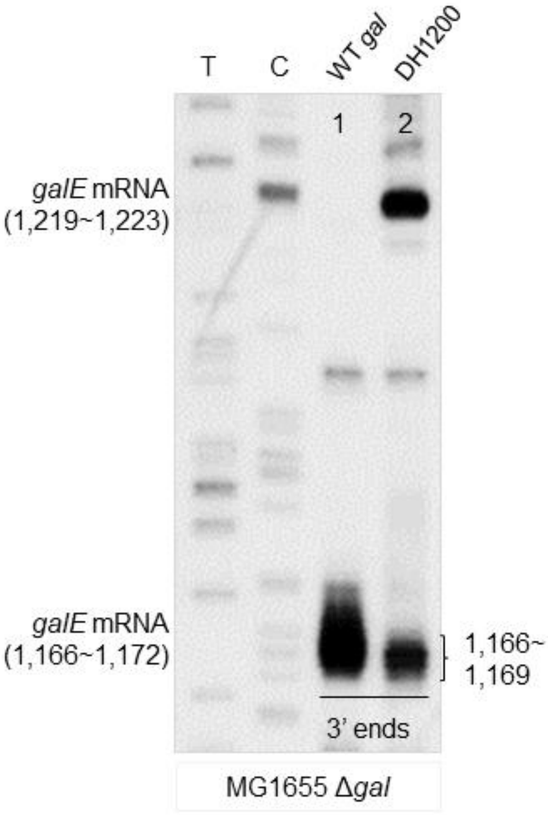
T
C
WT gal
DH1200
1
2
galE mRNA
(1,219~1,223)
galE mRNA
(1,166~1,172)
1,166~
1,169
3’ ends
MG1655 Δgal

**Fig. S2**

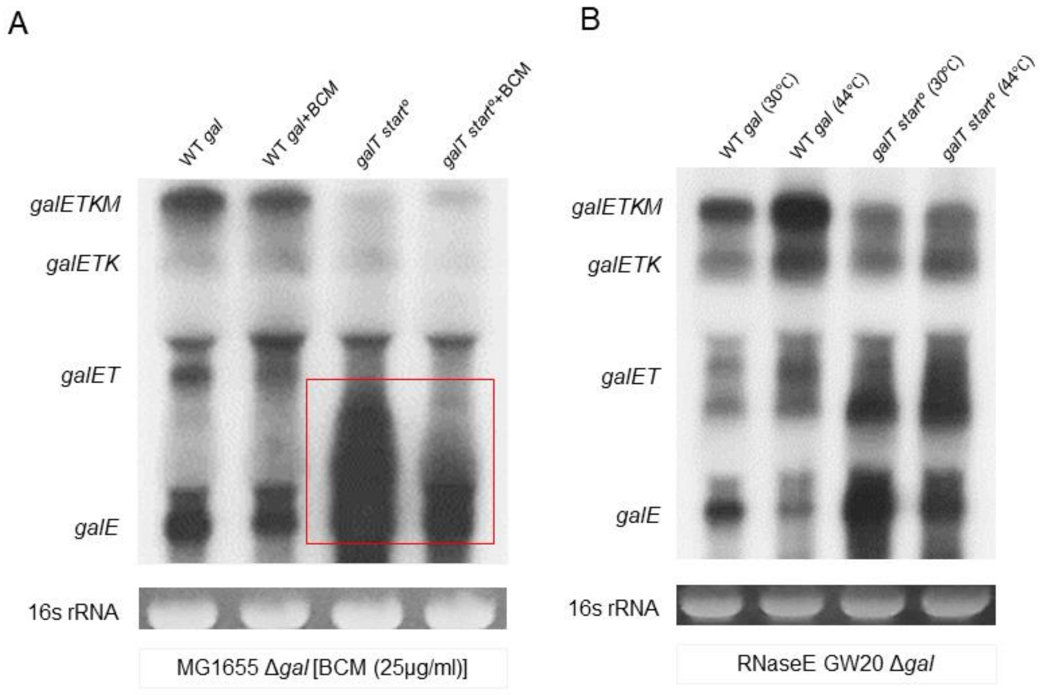
A
WT gal
WT gal+BCM
galT start°
galT start°+BCM
galETKM
galETK
galET
galE
16s rRNA
MG1655 Δgal [BCM (25μg/ml)]
B
WT gal (30°C)
WT gal (44°C)
galT start° (30°C)
galT start° (44°C)
galETKM
galETK
galET
galE
16s rRNA
RNaseE GW20 Δgal

**Fig. S3**

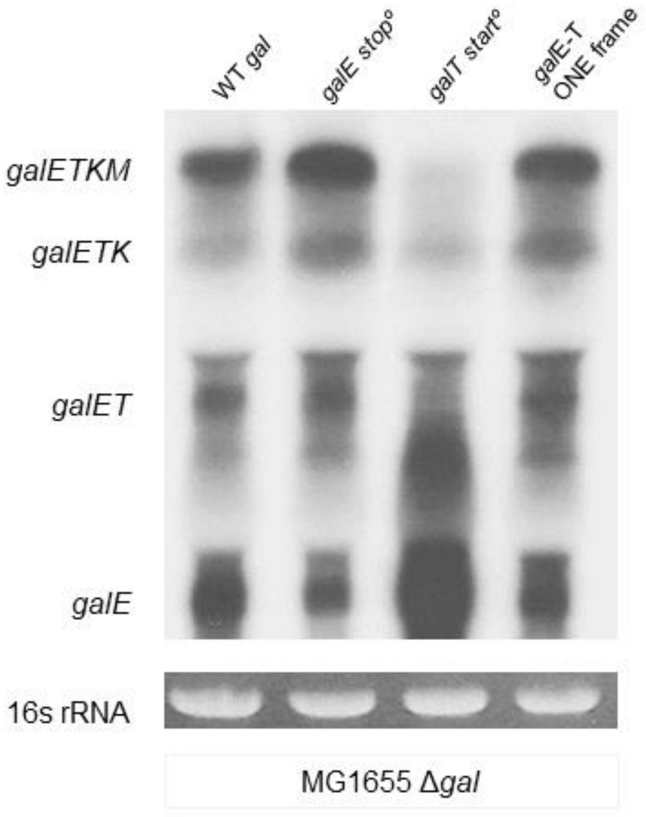
WT gal
galE stop°
galT start°
galE-T
ONE frame
galETKM
galETK
galET
galE
16s rRNA
MG1655 Δgal

**Fig. S4**

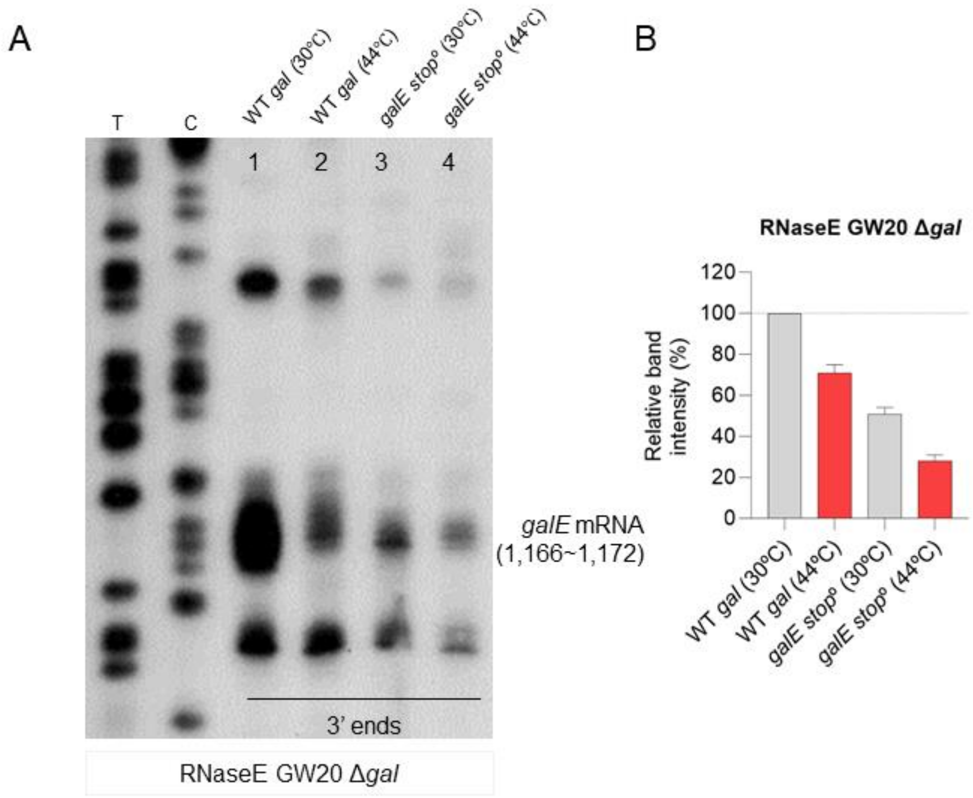
A
T
C
WT gal (30°C)
WT gal (44°C)
galE stop° (30°C)
galE stop° (44°C)
1
2
3
4
galE mRNA
(1,166~1,172)
3' ends
RNaseE GW20 Δgal
B
RNaseE GW20 Δgal
Relative band intensity (%)
120
100
80
60
40
20
0
WT gal (30°C)
WT gal (44°C)
galE stop° (30°C)
galE stop° (44°C)

**Table. S1**

| Primer name | Primer sequences (5′→3′) | Usage |
|---|---|---|
| *galBamHI-R* | CCGGATCCGGTGATTTGAACAATATGAG | Cloning |
| *galHindIII-F* | CACCGTTTATGGCGATCAGCCC | |
| *galMluI-R* | GCGTTTTCAGTCAGTATATGACG | |
| *EHMM2-F* | CGGCCCCTGGCAGGGGGCGCAGGAAA | Site-directed mutagenesis |
| *EHMM5-F* | CGCGGCCTGGCAGGGGGCGCAGGAAA | |
| *ENO-stop-R* | GGTCGTTCCTTTATCCGGATATC | |
| *TNO-start-F* | TAAGGAACGACCAAAACGCAA | |
| *ET-one frame* | AAAGGAACGACCAAAACGCAA | |
| *DH1200aatII-F* | GTTACCTGCGCACGATCCAGACGTC | |
| *DH1200aatII-R* | ACATTACCTGCGCAGAGGAAGACGTC | |
| *850 MluI-R* | CCATTACGCGTATGGTATGAAATAACC | |
| *850 PstI-R* | CAAATTCACGCCTGCAGGCGCCTGATT | |
| *E1-F* | ATGAGAGTTCTGGTTACCGGTGGTAG | E-probe generation for Northern blot |
| *E1-R* | TGGGCTTTTTTGCAGATCGGTGAGGA | |
| *3RP* | AGCATGCGGCCGCTAAGAAC | 3' RACE – RT and PCR primers |
| *3′RNA Oligo* | UUCACUGUUCUUAGCGGCCGCAUGCU | |
| *E3-F* | CATCGCCCAGGTTGCTGTAG | |
| *NewT6_1123-F* | TTTCACCGCACCGCGCTAA | 3' RACE extension primer |
| *T1, T-ext1,T6-F* | ATGACGCAATTTAATCCCGTTGATCATC | |
| *5s-F* | GAGAGTAGGGAACTGCCA | 5′ RACE PCR primer |
| *K2-R* | AGCCTACAAACTGGTTTTCTGCTTCC | |
| *M2-1-R* | CATCTGAACTCAGGGCAAACA | |
| *EText-R* | AGAATCCATTGCCCGGTGAG | 5`RACE extension primer |
| *TKext-R* | ATGGTGTGAGTGGCAGGGTA | |
| *KMext-R* | TGCCAGTGCGGGAGTTTCGT | |
